# Comparative untargeted metabolome analysis of ruminal fluid and feces of Nelore steers (*Bos indicus*)

**DOI:** 10.1038/s41598-021-92179-y

**Published:** 2021-06-17

**Authors:** Jessica Moraes Malheiros, Banny Silva Barbosa Correia, Caroline Ceribeli, Daniel Rodrigues Cardoso, Luiz Alberto Colnago, Stanislau Bogusz Junior, James Mark Reecy, Gerson Barreto Mourão, Luiz Lehmann Coutinho, Julio Cesar Pascale Palhares, Alexandre Berndt, Luciana Correia de Almeida Regitano

**Affiliations:** 1 Embrapa Southeast Livestock, São Carlos, São Paulo Brazil; 2 Chemistry Institute of São Carlos, University of São Paulo/USP, São Carlos, São Paulo Brazil; 3 Embrapa Instrumentation, São Carlos, São Paulo Brazil; 4 Department of Animal Science, Iowa State University, Ames, IA USA; 5 Department of Animal Science, University of São Paulo/ESALQ, Piracicaba, São Paulo Brazil

**Keywords:** Metabolic pathways, Metabolomics, Metabolomics, Animal breeding

## Abstract

We conducted a study to identify the fecal metabolite profile and its proximity to the ruminal metabolism of Nelore steers based on an untargeted metabolomic approach. Twenty-six Nelore were feedlot with same diet during 105 d. Feces and rumen fluid were collected before and at slaughter, respectively. The metabolomics analysis indicated 49 common polar metabolites in the rumen and feces. Acetate, propionate, and butyrate were the most abundant polar metabolites in both bio-samples. The rumen presented significantly higher concentrations of the polar compounds when compared to feces (*P* < 0.05); even though, fecal metabolites presented an accentuated representability of the ruminal fluid metabolites. All fatty acids present in the ruminal fluid were also observed in the feces, except for C20:2n6 and C20:4n6. The identified metabolites offer information on the main metabolic pathways (higher impact factor and *P* < 0.05), as synthesis and degradation of ketone bodies; the alanine, aspartate and glutamate metabolisms, the glycine, serine; and threonine metabolism and the pyruvate metabolism. The findings reported herein on the close relationship between the ruminal fluid and feces metabolic profiles may offer new metabolic information, in addition to facilitating the sampling for metabolism investigation in animal production and health routines.

## Introduction

Brazil has the world’s second largest commercial beef cattle herd, estimated at 244 million head^1^. The Nelore breed (*Bos indicus*) is the most important beef cattle breed in the country, representing alone or in crosses around 80% of the national herd, mostly due to its adaptability to tropical climate and greater parasite resistance^2,3^.

Rumen has a stable and dynamic microbial ecosystem, and the symbiotic relationship between the host and its microbiota plays a central role in food digestion, and provides energy for metabolic functions including growth^4–6^. The high density and diverse microbiome complex acts as an efficient system for converting plant cell wall biomass into microbial proteins, volatile fatty acids and gases^7–9^. Divergences in microbial populations can interfere with food degradation and with the composition and availability of metabolites in the rumen^10,11^. Furthermore, a wide range of metabolites that perform complex metabolic activities in rumen are also used by microorganisms for their own proliferation^12^.

In recent years, metabolomics has been used to clarify the underlying biological mechanisms that contribute towards the understanding of rumen microbial community structure, metabolic potential, and metabolic activity^13^, once metabolites are effectively the end products of complex reactions^14^. Consequently, ruminal fluid metabolites have shown promising results towards the understanding of biochemical rumen engineering^15–19^.

The rumen microbiota is partially represented in the fecal microbiota^20^, although scarce studies are available on fecal metabolites in cattle^21,22^. It is evident that it is easier to access fecal than rumen samples during animal production routines, so the identification of possible metabolic pathways and biomarkers in feces would have great potential for practical applications. In addition, considering that the rumen metabolism is specific for each breed, studies that investigate ruminal fluid and feces in the Nelore breed are required in order to increase our understanding and provide crucial biological information on the complex interactions between host genetics, gut microbiome and their metabolites. In this context, this study hypothesizes that rumen fluid and feces metabolite profiles share common biochemical pathways, where fecal metabolomics may also represent the host-microbiome co-metabolism. To this end, metabolomics fingerprinting of these two bio-samples from Nelore steers was performed using ^1^H NMR spectroscopy and GC-FID.

## Results

**Figure 1:**
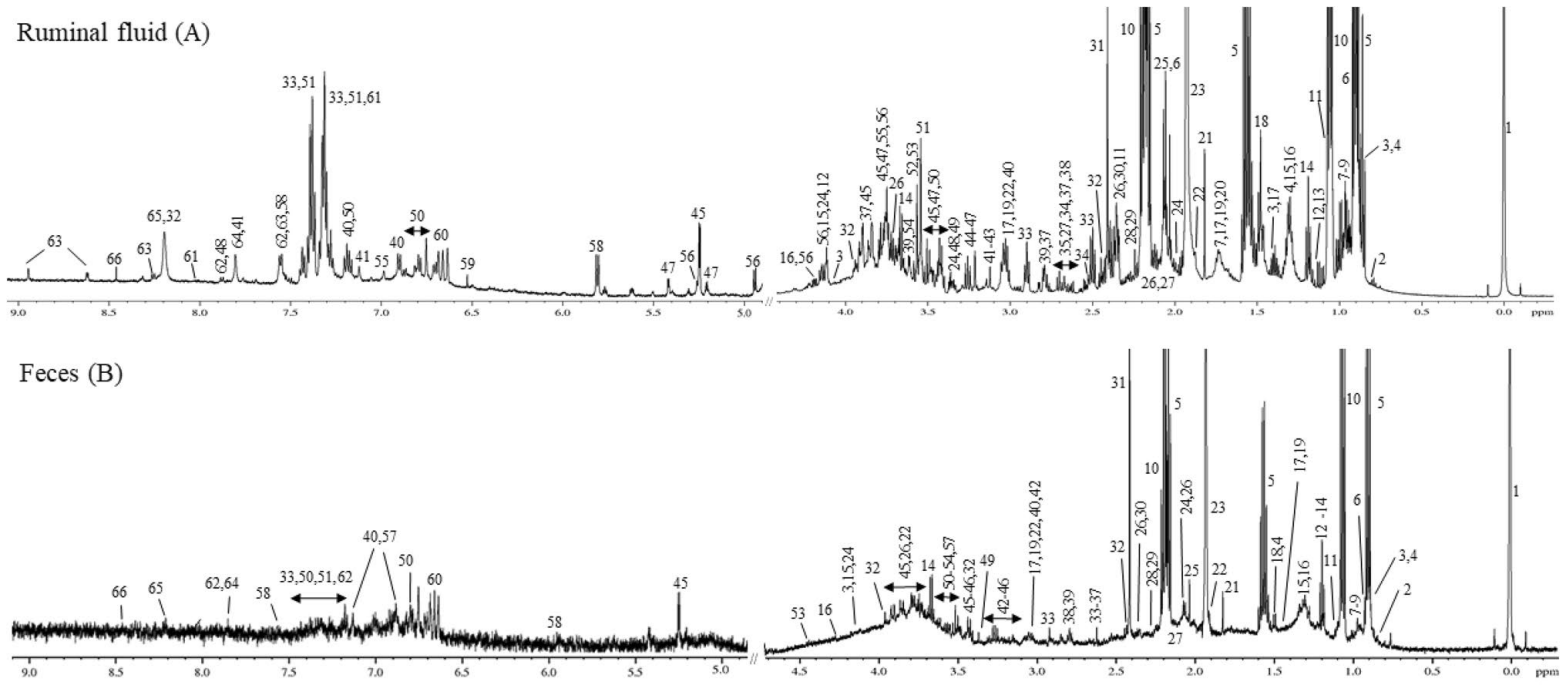
Representative ^1^H NMR spectrum of ruminal fluid (**A**) and feces (**B**) of Nelore steers. 1: TMSP-d4; 2: 2-Hydroxyisovalerate; 3: 2-Hydroxyvalerate; 4: Valerate; 5: Butyrate; 6: Isovalerate; 7: Leucine; 8: Isoleucine; 9: Valine; 10: Propionate; 11: Isobutyrate; 12: 3-Hydroxybutyrate; 13: Isopropanol; 14: Ethanol; 15: Lactate; 16: Threonine; 17: Lysine; 18: Alanine; 19: Cadaverine; 20: 4-Hydroxybutyrate; 21: Unknown metabolite; 22: Ornithine; 23: Acetate; 24: Proline; 25: Unknown metabolite; 26: Glutamate; 27: Methionine; 28: Acetone; 29: Acetoacetate; 30: Pyruvate; 31: Succinate; 32: Panthotenate; 33: 3-Phenylpropionate; 34: Citrate; 35: Methylamine; 36: β-Alanine; 37: Aspartate; 38: Dimethylamine; 39: Sarcosine; 40: Tyrosine; 41: Histidine; 42: Unknown metabolite; 43: N-nitrosodimethylamine; 44: Choline; 45: Glucose; 46: Unknown metabolite; 47: Maltose; 48: Caffeine; 49: Methanol; 50: 3-Hydroxyphenylacetate; 51: Phenylacetate; 52: Glycine; 53: 1,3-Dihydroxyacetone; 54: Glycerol; 55: 4-Hydroxy-3-methoxymandelate; 56: Ribose; 57: Unknown metabolite; 58: Uracil; 59: Fumarate; 60: Unknown metabolite; 61: Imidazole; 62: Benzoate; 63: Nicotinate; 64: Xanthine; 65: Hypoxanthine; 66: Formate.

Metabolites extracted from the rumen and fecal samples were analyzed by ^1^H NMR. The spectra were comprised by organic acids (34.5%), amino acids (27.6%), nucleic acids (8.6%), alcohol (8.6%), sugars (6.9%), amines (5.2%), and other compounds (8.6%) (Fig. 1). Fifty-eight polar metabolites were identified and quantified in ruminal fluid and 50 metabolites were present in the fecal samples. Comparisons between spectra revealed that the metabolite diversity of the rumen is slightly higher than the fecal samples, although 49 metabolites were observed in both samples. The derived organic acid (fumarate and 4-Hydroxybutyrate), amino acids (histidine), sugars (maltose and ribose) and other compounds (caffeine, nicotinate, 4-Hydroxy-3-methoxymandelate and imidazole) were observed only in the ruminal fluid.

**Figure 2:**
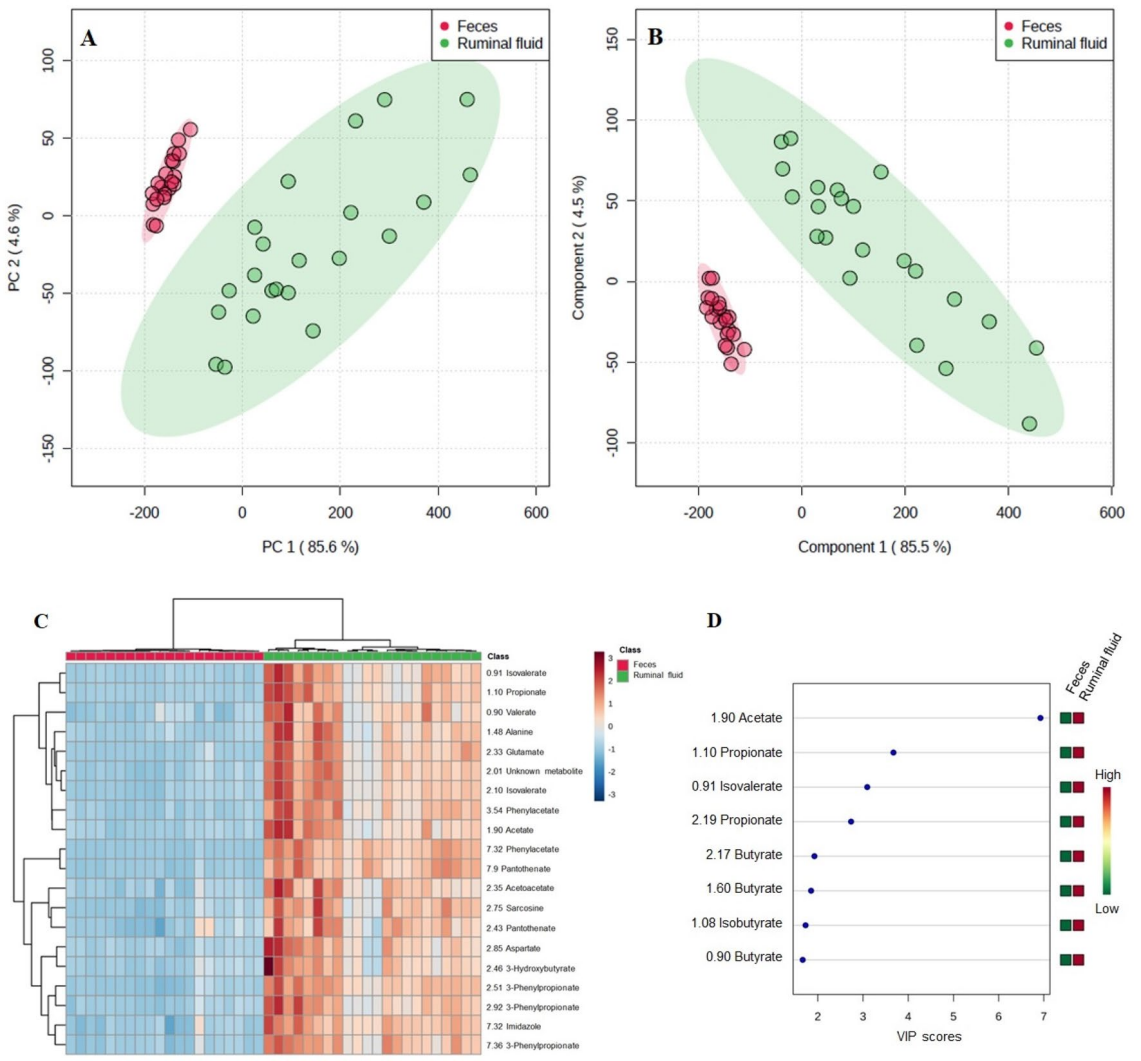
Ruminal fluid and feces metabolomic profile of the Nelore steers. (**A**) Principal component analysis for metabolites identified by ^1^H NMR (One data point represents one steer). (**B**) Partial least square-discriminant analysis for ^1^H NMR spectrum metabolites identified (One data point represents one steer). (**C**) Hierarchical clustering analysis (heatmap) of metabolomic differences between ruminal fluid and feces by T-test/ANOVA (chemical spectrum shift—ppm). (**D**) Top 5 metabolites (chemical spectrum shift—ppm) selected by VIP score (VIP > 1.5).

Principal component analysis (PCA) and partial least squares discriminant analysis (PLS-DA) were applied to the ^1^H NMR spectra data to visualize differences in the ruminal fluid and fecal metabolite profiles. The scores plot (Component 1/Component 2) of both PCA and PLS-DA indicated a 90% coverage of the observed sample set by the first two principal components being the major variation in the sample set explained by the first principal component (Fig. 2A,B). Overall, the analysis revealed a clear group separation between the ruminal fluid and fecal metabolites. For the first PLS-DA component, descriptive statistics from model fitting by accuracy, estimates of the goodness of fit (R^2^), and estimates of goodness prediction (Q^2^) were as follows: accuracy = 0.90, R^2^ = 0.65, and Q^2^ = 0.61, and accuracy = 1.0, R^2^ = 0.93, and Q^2^ = 0.91 for the second component. The differences also can be clearly observed in the clusters generated in the heatmap plot by the hierarchical clustering analysis (HCA) (Fig. 2C).

To define which compounds were influential metabolites in the PLS-DA model, a feature selection based on VIP scores was performed (Fig. 2D). The procedure allowed for the identification of the variables that were most important for the separation of both samplings. Accordingly, five organic acid derived compounds [butyrate (0.87; 1.55; 2.15 ppm), isobutyrate (1.03 ppm), propionate (2.19; 1.07 ppm); isovalerate (0.91 ppm) and acetate (1.91 ppm)] presented VIP > 1.5 and were identified as key lineages for separating the ruminal and fecal metabolic profiles.

The most abundant polar metabolites present in ruminal fluid and feces were volatile/short chain fatty acids including acetate, propionate, butyrate and amino acids, followed by the organic acids 2-hydroxyvalerate, isobutyrate, valerate and isovalerate in ruminal fluid (Table 1). In fecal samples, high concentrations of succinate, valerate, isobutyrate and alanine metabolites were also observed. Rumen presented significantly higher concentrations of most polar metabolites when comparing to samples collected from the rectal ampulla (*P* < 0.05). Among these metabolites, acetate in feces decreased 1.7-fold when compared to ruminal fluid. Furthermore, propionate and butyrate in the ruminal fluid were 1.8 and 1.3-fold higher than in fecal samples, respectively. In addition, no significant difference was found in the concentration of 11 metabolites (*P* > 0.05), such as acetoacetate, formate, benzoate, proline, valine, and others between the two different bio-samples. Some compounds, like lactate, pyruvate, threonine, pantothenate, and others were present in higher concentrations in feces when compared to ruminal fluid (*P* < 0.05).

**Table 1:** Concentrations of ruminal fluid and fecal metabolites of Nelore steers determined by ^1^H RMN and GC-FID.

Polar metabolites	Formula	ID^a^	Ruminal fluid (µM)^b^	Feces (µM)^b^	*P*^*c*^
**Derived organic acid**					
Acetate	C_2_H_4_O_2_	BMDB0000042	14,694.99 ± 5075.59	8505.42 ± 2661.88	< .0001
Propionate	C_3_H_6_O_2_	BMDB0000237	3588.24 ± 1994.33	1994.33 ± 672.08	< .0001
Butyrate	C_4_H_8_O_2_	BMDB0000039	2495.09 ± 1045.54	1895.90 ± 685.20	0.02
2-Hydroxyvalerate	C_5_H_10_O_3_	BMDB0001863	617.68 ± 191.82	239.65 ± 122.24	< .0001
Isobutyrate	C_4_H_8_O_2_	BMDB0001873	539.79 ± 159.51	314.05 ± 117.67	0.02
Valerate	C_5_H_10_O_2_	BMDB0000892	413.27 ± 102.37	326.15 ± 142.34	0.02
Isovalerate	C_5_H_10_O_2_	BMDB0000718	389.69 ± 122.48	226.60 ± 75.87	< .0001
Phenylacetate	C_8_H_8_O_2_	BMDB0000209	316.42 ± 62.91	33.16 ± 14.77	< .0001
Succinate	C_4_H_6_O_4_	BMDB0000254	241.22 ± 64.71	423.79 ± 133.93	< .0001
4-Hydroxybutyrate	C_4_H_8_O_3_	BMDB0000710	177.28 ± 58.79		< .0001
3-Phenylpropionate	C_9_H_10_O_2_	BMDB0000764	146.99 ± 48.83	82.34 ± 42.22	< .0001
3-Hydroxybutyrate	C_4_H_8_O_3_	BMDB0000357	128.43 ± 38.30	79.08 ± 31.53	< .0001
2-Hydroxyisovalerate	C_5_H_10_O_3_	BMDB0000407	94.72 ± 41.36	35.99 ± 12.97	< .0001
Lactate	C_3_H_6_O_3_	BMDB0001311	79.88 ± 26.32	144.61 ± 64.56	< .0001
3-Hydroxyphenylacetate	C_8_H_8_O_3_	BMDB0000440	79.67 ± 27.80	144.41 ± 83.93	0.001
Acetoacetate	C_4_H_6_O_3_	BMDB0000060	39.13 ± 12.44	35.05 ± 14.01	0.306
Citrate	C_6_H_8_O_7_	BMDB0000094	21.02 ± 5.58	15.22 ± 6.46	0.002
Pyruvate	C_3_H_4_O_3_	BMDB0000243	20.57 ± 6.52	37.94 ± 17.56	< .0001
Formate	CH_2_O_2_	BMDB0000142	6.77 ± 3.90	9.07 ± 3.76	0.654
Benzoate	C_7_H_6_O_2_	BMDB0062037	4.04 ± 2.75	5.01 ± 2.48	0.511
Fumarate	C_4_H_4_O_4_	BMDB0000134	3.02 ± 1.98		< .0001
**Amino acid**					
Glutamate	C_5_H_9_NO_4_	BMDB0003339	1111.30 ± 275.75	603.29 ± 271.84	< .0001
Alanine	C_3_H_7_NO_2_	BMDB0000161	273.25 ± 75.66	242.52 ± 98.00	0.679
Ornithine	C_5_H_12_N_2_O_2_	BMDB0000214	174.09 ± 56.87	103.25 ± 46.93	< .0001
Isoleucine	C_6_H_13_NO_2_	BMDB0000172	170.68 ± 73.03	98.62 ± 42.08	0.0002
Glycine	C_2_H_5_NO_2_	BMDB0000123	133.82 ± 32.78	70.86 ± 23.54	< .0001
Proline	C_5_H_9_NO_2_	BMDB0000162	130.04 ± 46.17	136.53 ± 58.16	0.679
Aspartate	C_4_H_7_NO_4_	BMDB0000191	125.17 ± 26.15	199.08 ± 32.17	< .0001
Leucine	C_6_H_13_NO_2_	BMDB0000687	114.33 ± 39.23	63.40 ± 26.59	< .0001
Valine	C_5_H_11_NO_2_	BMDB0000883	114.10 ± 36.42	133.70 ± 35.06	0.216
Threonine	C_4_H_9_NO_3_	BMDB0000167	74.46 ± 15.81	139.52 ± 38.42	< .0001
Tyrosine	C_9_H_11_NO_3_	BMDB0000158	55.41 ± 15.26	59.54 ± 28.15	0.541
Histidine	C_5_H_9_N_3_	BMDB0000177	32.45 ± 7.53		< .0001
Sarcosine	C_3_H_7_NO_2_	BMDB0000271	30.83 ± 8.49	66.52 ± 10.37	0.0002
Methionine	C_5_H_11_NO_2_S	BMDB0000696	29.94 ± 11.32	36.12 ± 16.98	0.156
Lysine	C_6_H_14_N_2_O_2_	BMDB0000182	18.99 ± 5.62	14.85 ± 6.45	0.02
β-Alanine	C_3_H_7_NO_2_	BMDB0000056		11.56 ± 5.36	< .0001
**Sugars**					
Maltose	C_12_H_22_O_11_	BMDB0000163	225.30 ± 85.31		< .0001
Glucose	C_6_H_12_O_6_	BMDB0000122	100.80 ± 40.24	226.73 ± 60.29	< .0001
Ribose	C_5_H_10_O_5_	BMDB0000283	73.59 ± 32.34		< .0001
1,3-Dihydroxyacetone	C_3_H_6_O_3_	BMDB0001882	33.16 ± 13.95	31.08 ± 13.89	0.618
**Amines**					
Cadaverine	C_5_H_14_N_2_	BMDB0002322	46.70 ± 14.64	40.41 ± 18.09	0.205
Dimethylamine	C_2_H_7_N	BMDB0000087	37.06 ± 12.41	5.87 ± 3.05	< .0001
Methylamine	CH_5_N	BMDB0000164	2.72 ± 1.04	23.33 ± 8.77	< .0001
**Nitrosamines**					
N-Nitrosodimethylamine	C_2_H_6_N_2_O	BMDB0063671	24.22 ± 5.39	15.26 ± 7.15	0.02
**Quaternary ammonium salts**					
Choline	C_5_H_14_NO	BMDB0000097	15.88 ± 6.07	5.33 ± 2.93	< .0001
**Nucleotide/Purines/ Pyridine**					
Hypoxanthine	C_5_H_4_N_4_O	BMDB0000157	96.92 ± 30.86	13.73 ± 8.38	< .0001
Uracil	C_4_H_4_N_2_O_2_	BMDB0000300	38.84 ± 10.32	2.08 ± 0.86	< .0001
Xanthine	C_5_H_4_N_4_O_2_	BMDB0000292	32.67 ± 12.16	6.47 ± 2.65	< .0001
Caffeine	C_8_H_10_N_4_O_2_	BMDB0001847	22.87 ± 9.44		< .0001
Nicotinate	C_6_H_5_NO_2_	BMDB0001488	8.17 ± 5.77		< .0001
**Alkaloid**					
Imidazole	C_3_H_4_N_2_	BMDB0001525	15.78 ± 5.43		< .0001
**Alcohol**					
Ethanol	C_2_H_6_O	BMDB0000108	271.25 ± 91.39	412.06 ± 107.04	0.004
Methanol	CH_4_O	BMDB0001875	78.70 ± 23.69	86.00 ± 30.74	0.910
Pantothenate	C_9_H_17_NO_5_	BMDB0000210	71.65 ± 17.56	152.32 ± 65.58	< .0001
Isopropanol	C_3_H_8_O	BMDB0000863	37.86 ± 9.63	99.31 ± 24.11	< .0001
Glycerol	C_3_H_8_O_3_	BMDB0000131	28.88 ± 8.69	55.33 ± 15.26	< .0001
**Ketones**					
Acetone (or Propanone)	C_3_H_6_O	BMDB0001659	14.95 ± 4.37	22.46 ± 6.43	0.009
**Methoxyphenol**					
4-Hydroxy-3-methoxymandelate	C_9_H_10_O_5_	BMDB0000291	80.86 ± 27.34		< .0001

**Apolar metabolites**	**Formula**	**ID**^**a**^	**Ruminal fluid (mg/g)**^**b**^	**Feces (mg/g)**^**b**^	***P***^***c***^
**Fatty acids**					
C6:0	C_6_H_12_O_2_	BMDB0000535	0.054 ± 0.01	1.34 ± 0.61	< .0001
C8:0	C_8_H_16_O_2_	BMDB0000482	0.24 ± 0.09	0.43 ± 0.12	0.045
C10:0	C_10_H_20_O_2_	BMDB0000511	0.20 ± 0.08	1.16 ± 0.58	< .0001
C11:0	C_11_H_22_O_2_	BMDB0000947	0.09 ± 0.01	0.40 ± 0.17	< .0001
C12:0	C_12_H_24_O_2_	BMDB0000638	2.18 ± 1.12	6.21 ± 2.43	< .0001
C13:0	C_13_H_26_O_2_	BMDB0000910	0.79 ± 0.05	1.46 ± 0.60	< .0001
C14:0	C_14_H_28_O_2_	BMDB0000806	16.13 ± 6.09	31.41 ± 14.06	0.0008
C14:1n5	C_14_H_26_O_2_	BMDB0002000	0.06 ± 0.01	0.16 ± 0.05	0.0473
C15:0	C_15_H_30_O_2_	BMDB0000826	15.59 ± 3.55	14.24 ± 6.15	0.6666
C16:0	C_16_H_32_O_2_	BMDB0000220	353.48 ± 81.36	340.14 ± 142.35	0.8556
C16:1n7	C_16_H_30_O_2_	BMDB0012328	5.37 ± 2.13	3.91 ± 1.92	0.2486
C17:0	C_17_H_34_O_2_	BMDB0002259	9.77 ± 2.55	13.63 ± 6.39	0.0998
C18:0	C_18_H_36_O_2_	BMDB0000827	1174.44 ± 217.56	1412.70 ± 305.78	0.3686
C18:1n9	C_18_H_34_O_2_	BMDB0000207	26.84 ± 10.62	40.09 ± 17.51	0.0992
C18:2n6	C_18_H_32_O_2_	BMDB0109638	45.60 ± 17.84	103.70 ± 45.87	0.0003
C18:3n6	C_18_H_30_O_2_	BMDB0003073	3.47 ± 1.70	7.43 ± 3.48	0.0008
C20:0	C_20_H_40_O_2_	BMDB0109637	12.82 ± 3.43	18.40 ± 3.35	0.059
C20:1n9	C_20_H_38_O_2_	BMDB0002231	0.87 ± 0.01	2.92 ± 0.81	< .0001
C20:2n6	C_20_H_36_O_2_	BMDB0005060	0.30 ± 0.03		< .0001
C20:4n6	C_20_H_32_O_2_	BMDB0001043	0.38 ± 0.04		< .0001
C22:0	C_22_H_44_O_2_	BMDB0000944	6.05 ± 1.44	16.37 ± 6.76	< .0001
C22:1n9	C_22_H_42_O_2_	BMDB0002068	2.46 ± 0.85	1.49 ± 0.96	0.0686
C22:2n6	C_22_H_40_O_2_	BMDB0109678	0.78 ± 0.12	3.10 ± 1.03	0.0016
C24:1n9	C_24_H_46_O_2_	BMDB0002368	0.15 ± 0.08	0.82 ± 0.34	0.0016

^a^ID Bovine Metabolome Database (BMDB).

^b^Mean ± Standard deviation.

^c^*P* correspond to the original P-value calculated from the enrichment analysis.

Apolar metabolites were extracted from the rumen and fecal samples and analyzed by GC-FID. The chromatograms indicated 22 fatty acids present in both bio-samples, except for C20:2n6 and C20:4n6, which were observed only in ruminal fluid. The apolar metabolites C18:0 and C16:0 were the most abundant in ruminal fluid and feces, followed by the C18:2n6, C18:1n9 and C14:0. Feces presented significantly higher concentrations of most fatty acids when compared to ruminal samples (*P* < 0.05). In addition, no significant difference between samples collected from the rumen and rectal ampulla was observed regarding C15:0 and C16:0 (*P* > 0.05).

**Figure 3:**
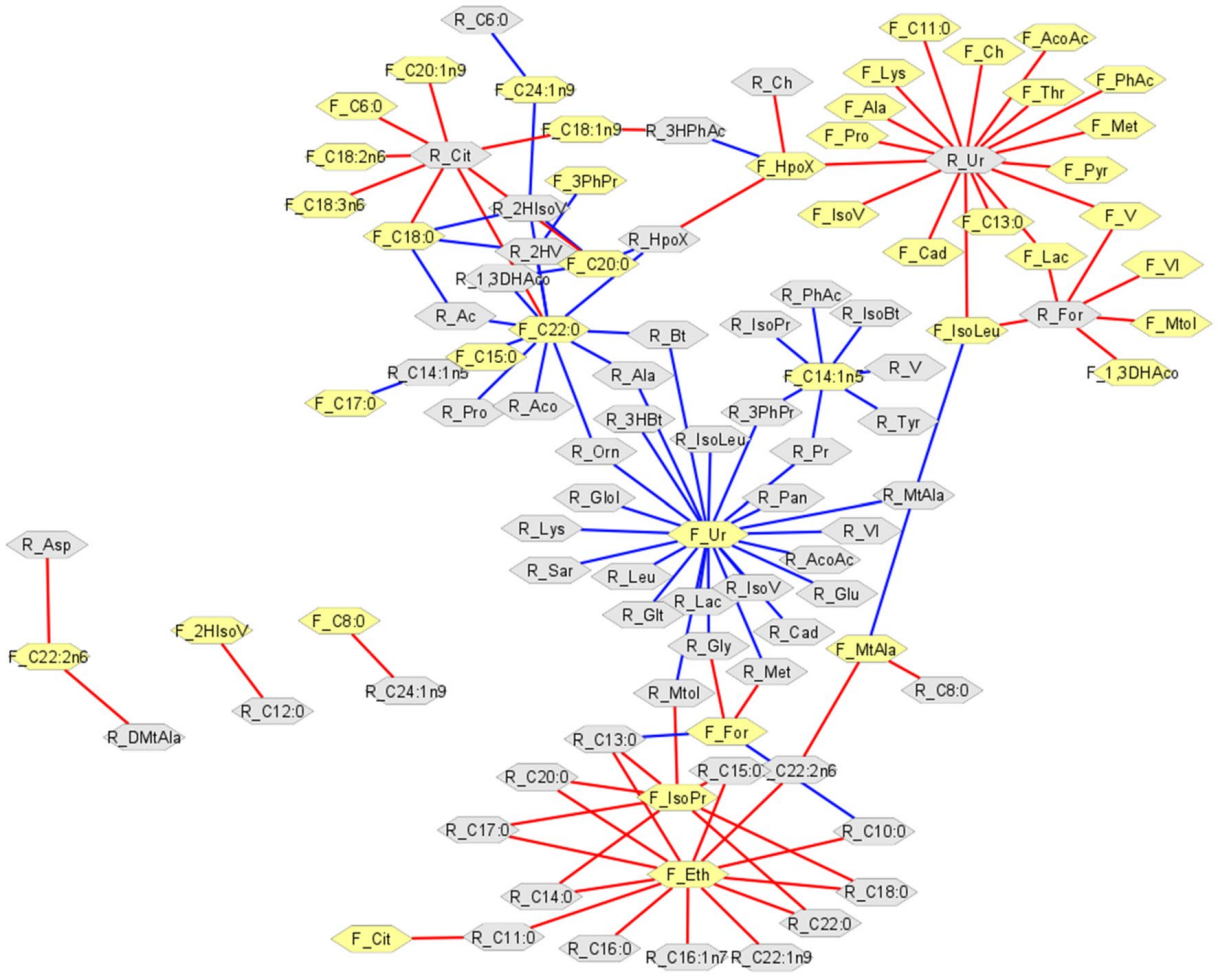
Correlation network between metabolic ruminal and feces profiles in Nelore steers (Spearman's correlation with P ≤ 0.05), carried out using the Cytoscape software. Gray hexagons represent ruminal fluid metabolites and yellow hexagons, fecal metabolites. Red lines correspond to negative correlations, whereas blue lines correspond to positive correlations between the analyzed bio-samples. The acronyms of each metabolite and correlation coefficient values are presented in Supplementary Table 1, 2.

A correlation network analysis was used to detect the comprehensive relationships between metabolic rumen fluid and feces profiles (Fig. 3). Of the 4,900 possible pairs analyzed, 122 displayed significant correlations (*P* ≤ 0.05) and were considered for the network construction. Among these, 60 presented positive and 62, negative, correlations (Supplementary Table 1, 2). The uracil metabolite found in ruminal fluid (R_Ur) presented a negative correlation with fecal metabolites, such as organic acid (pyruvate, valerate, and phenylacetate) and amino acids (threonine, and proline). The uracil in feces (F_Ur) was positively correlated to organic acids (butyrate, propionate, 3-Hydroxybutyrate, and 3-Phenylpropionate) and amino acids (glucose, ornithine, glutamate, valine, sarcosine, glycine, and leucine) in rumen. However, some ruminal fluid metabolites were correlated with fecal uracil, and the same fecal metabolites were correlated with uracil present in the ruminal fluid. Among these metabolites, are noteworthy to mention: organic acids (acetoacetate, isovalerate, and lactate) and amino acids (alanine, lysine, methionine, and isoleucine).

**Figure 4:**
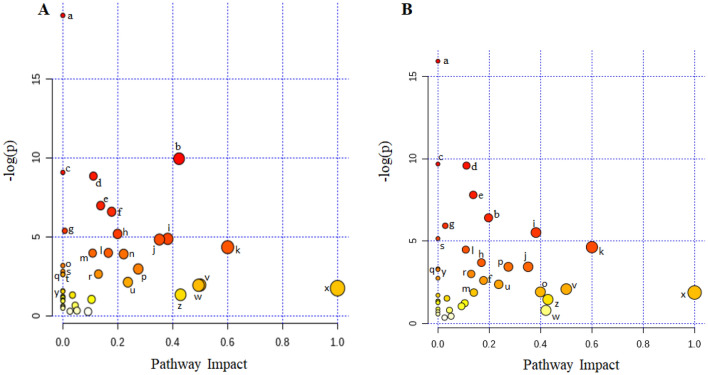
Pathway analysis of ruminal fluid (**A**) and feces (**B**) polar metabolites of Nelore steers. This analysis was undertaken using MetaboAnalyst 4.0 software according to the *Bos taurus* KEGG pathway database. Darker colored, larger areas of the bubbles represent more significant metabolite changes in the corresponding pathway. The letters indicate the pathways (see Table 2).

In order to further understand the usefulness of the detected metabolites by the ^1^H NMR method, we performed pathway analysis to associate the metabolites to their corresponding pathways. The functional analysis of ruminal and fecal metabolites indicated that most metabolites were involved in more than one pathway (Fig. 4). In addition, the same metabolic pathways were observed for both ruminal fluid and feces (*P* < 0.05), except for the tyrosine metabolism, histidine metabolism, and beta-alanine metabolism which were uniquely significant in the rumen (Table 2).

**Table 2:** Results from ruminal fluid and feces pathway analysis of Nellore steers.

Letter^a^	Pathway name	Total cmpd^b^	Ruminal fluid				Feces			
			Hits^c^	*P*^*d*^	− log(p)^e^	Impact^f^	Hits^a^	*P*^*d*^	− log(p)^e^	Impact^f^
a	Aminoacyl-tRNA biosynthesis	48	13	0.01	19.04	0.00	11	0.01	15.93	0.00
b	Alanine, aspartate and glutamate metabolism	28	7	0.01	9.95	0.42	5	0.01	6.42	0.20
c	Valine, leucine and isoleucine biosynthesis	8	4	0.01	9.08	0.00	4	0.01	9.69	0.00
d	Butanoate metabolism	15	5	0.01	8.85	0.11	5	0.01	9.59	0.11
e	Glyoxylate and dicarboxylate metabolism	32	6	0.01	6.98	0.14	6	0.01	7.81	0.14
f	Arginine biosynthesis	14	4	0.01	6.59	0.18	4	0.03	3.44	0.27
g	Pantothenate and CoA biosynthesis	19	4	0.01	5.38	0.01	4	0.01	5.94	0.03
h	Citrate cycle (TCA cycle)	20	4	0.01	5.18	0.20	3	0.02	3.70	0.17
i	Glycine, serine and threonine metabolism	34	5	0.01	4.87	0.38	5	0.01	5.53	0.38
j	Pyruvate metabolism	22	4	0.01	4.83	0.35	3	0.03	3.44	0.35
k	Synthesis and degradation of ketone bodies	5	2	0.01	4.34	0.60	2	0.01	4.64	0.60
l	Tyrosine metabolism	42	5	0.02	3.98	0.17	3	0.15	1.88	0.14
m	Glutathione metabolism	28	4	0.02	3.97	0.11	4	0.01	4.49	0.11
n	Histidine metabolism	16	3	0.02	3.91	0.22	1	0.41	0.89	0.00
o	beta-Alanine metabolism	21	3	0.04	3.18	0.00	2	0.15	1.92	0.40
p	Arginine and proline metabolism	38	4	0.05	2.96	0.27	2	0.07	2.62	0.18
q	Valine, leucine and isoleucine degradation	40	4	0.06	2.81	0.00	4	0.04	3.28	0.00
r	Glycolysis / Gluconeogenesis	26	3	0.07	2.64	0.13	3	0.05	3.01	0.13
s	Phenylalanine metabolism	12	2	0.07	2.63	0.00	3	0.01	5.16	0.00
t	Neomycin, kanamycin and gentamicin biosynthesis	2	1	0.07	2.60	0.00	1	0.06	2.75	0.00
u	Glycerolipid metabolism	16	2	0.12	2.12	0.24	2	0.09	2.38	0.24
v	Phenylalanine, tyrosine and tryptophan biosynthesis	4	1	0.14	1.95	0.50	1	0.12	2.09	0.50
w	Starch and sucrose metabolism	18	2	0.15	1.93	0.49	1	0.45	0.80	0.42
x	D-Glutamine and D-glutamate metabolism	5	1	0.18	1.74	1.00	1	0.15	1.88	1.00
y	Propanoate metabolism	23	2	0.21	1.54	0.00	3	0.04	3.33	0.00
z	Taurine and hypotaurine metabolism	8	1	0.27	1.33	0.43	1	0.23	1.46	0.43

^a^Letter corresponds to the information shown in Fig. 3.

^b^Total cmpd corresponds to total number of compounds in the pathway.

^c^Hits correspond to the actually matched number from the user uploaded data.

^d^*P* correspond to the original P-value calculated from the enrichment analysis.

^e^− log(p) correspond to the P-value logarithm.

^f^Impact correspond to the pathway impact value calculated from pathway topology analysis.

The results of the enrichment analysis showed that pantothenate and aminoacyl-tRNA biosynthesis; alanine, aspartate and glutamate metabolism; valine, leucine and isoleucine biosynthesis; butanoate metabolism; glyoxylate and dicarboxylate metabolism; arginine biosynthesis; pantothenate and CoA biosynthesis; citrate cycle (TCA cycle); glycine, serine and threonine metabolism; pyruvate metabolism; synthesis and degradation of ketone bodies; and glutathione metabolism were significantly enriched (*P* < 0.05) in both bio-samples. Among these, the pathways with highest impact were synthesis and degradation of ketone bodies; alanine, aspartate and glutamate metabolism; glycine, serine and threonine metabolism; and pyruvate metabolism.

## Discussion

In the present study, we investigated the metabolome of two areas of the gastrointestinal tract of Nelore steers, rumen and fecal ampulla, and the relationship between these environments using ^1^H NMR and GC-FID. To date, the ruminal and fecal metabolome of Nelore cattle had not been described in the literature. The ruminal fluid involves hundreds of microorganisms, which interact with each other and with the host, while also degrading plant material^17^. The metabolic composition of feces can aid in clarifying this complex interplay between ruminants and their rumen ecosystem^21^, since this biological matrix contains information on the host, microbiota and feed components. Our metabolome data revealed a wide diversity of metabolites in ruminal fluid and feces due to the extraordinary activity of microorganisms, as previously described^12,21,23,24^. However, the comparison between rumen and the rectal ampulla also revealed that the Nelore cattle ruminal fluid metabolome is a little more diverse compared to the feces metabolome. Possibly certain metabolites were not detected in feces, due to high rumen solubility and degradability, although most metabolites (n = 49) have been observed in both bio-samples. These results confirm that the rumen metabolism is closely associated with the fecal metabolism.

In the present study, we observed a wide variety of compounds involved in multiple biochemical processes in rumen. The detected organic acids and amino acids indicate a high diversity of compounds in both bio-samples. The organic acids are fermentation products in rumen^25^. Furthermore, many microorganisms in rumen contribute to the degradation of dietary protein into amino acids, which are a wide range of important compounds for animal body maintenance and performance, besides being required for microbial growth^15^. Additionally, the microbiota is capable of amino acid synthesis through the use of acetate, propionate, among others to obtain carbon and nitrogen compounds as nitrogen sources^26–28^. In a recent study, Foroutan et al.^24^ described the ruminal fluid from *Bos taurus* cattle using NMR, partially agreeing with the metabolic profile reported herein for *Bos indicus*. These differences are mainly due to breed, environment, handling and nutrition, as the identification and concentration of many ruminal fluid metabolites are strongly affected by these traits.

Some signals in the ruminal fluid and fecal spectra could not be identified (unknown metabolites) in the used database. According to Almeida et al.^17^, uncharacterized molecules may have multiple origins and may be secreted by microorganisms, plants, and the host. Thus, further exploration of the rumen and fecal environment is required to completely understand the metabolome dataset and characterize metabolites not yet documented.

One particularly interesting point is the fact that the ruminal fluid of Nelore steers contains certain metabolites not observed in the fecal metabolome. Some of these metabolites can be quickly utilized during rumen fermentation, being degraded, catalyzed or metabolized, and thus not being detected in feces. It may be assumed, for example, that in the present study some dietary nutrients were degraded to maltose and later catalyzed by maltase and degraded to glucose in the rumen. As maltose has an energetic function, we propose it may have been rapidly used by microorganisms, and, therefore, was not able to accumulate in rumen, preventing its detection in fecal samples. Fumarate was also detected only in ruminal fluid, possibly due to the reduction of this metabolite into succinate in the rumen, which resulted in higher succinate concentrations in the fecal samples. Histidine is well-known to be extensively degraded by rumen microorganisms for use in microbial growth^29^. Ribose can be produced in the rumen from the degradation of nucleic acids from food sources or from dead bacterial cells, although it does not accumulate in the rumen, being rapidly metabolized by certain microorganisms.

Some metabolites (maltose, histidine, ribose, caffeine, nicotinate, imidazole, 4-Hydroxybutyrate and 4-Hydroxy-3-methoxymandelate metabolites) were observed for the first time in our study. Foroutan et al.^24^ evaluated the chemical composition of the ruminal fluid of *Bos taurus* through different technological platforms, and did not observe maltose, caffeine, imidazole, 4-Hydroxybutyrate and 4-Hydroxy-3-methoxymandelate metabolites. Similarly, Saleem et al.^12^ did not detect the 4-Hydroxy-3-methoxymandelate metabolite. O'Callaghan et al.^30^ reported the same for the rumen fluid of cows submitted to different pasture feeding systems. Recently, a study assessing the fecal metabolome in Jiulong Yak bovines using ^1^H NMR also reported the absence of these metabolites (4-Hydroxybutyrate, histidine, maltose, ribose, caffeine, nicotinate, 4-Hydroxy-3-methoxymandelate and imidazole), except for fumarate^21^. Thus, the differences between ruminal fluid metabolic profiles may be attributed to different breed, nutritional interventions and compound availability, which can intervene as elementary factors in the biochemical engineering of rumen and reflect directly on fecal metabolites.

The present study also demonstrated the nicotinate was detected only in rumen. While nicotinate is widely distributed in feed, this compound can have limited availability in rumen^31^, yet several bacterial species have the ability to synthesize this compound^30^. Nicotinate is intimately involved in the energy metabolism and may play a significant role in urea synthesis, which is a non-protein N source for rumen microorganisms^31^.

A chemometrics analysis within a biological context indicates that the compounds observed in the feces spectra were significantly altered when compared to rumen fluid. This clustering is mainly due to metabolite quantification, where the rumen contained higher concentrations of most compounds. The alteration of the concentration of a given ruminal metabolite may be correlated to the concentration of other metabolites, since compounds do not exist independently^32^, and fecal metabolites correlations may partially reflect the metabolic processes that occur in rumen.

As expected, the most abundant molecules were acetate, propionate and butyrate, representing the main plant material catabolism products obtained by the rumen microbiome. In this regard, these metabolites were also represented in feces by a portion of rumen fluid compound concentrations. The result concerning these metabolites corroborate the findings reported by Eom et al.^23^ for dairy cattle ruminal fluid. Foroutan et al.^24^ also observed these metabolites as the most abundant in *Bos taurus* ruminal fluid, although, butyrate was present in higher concentrations compared to propionate. According to Saleem et al.^12^, starch-rich diets increase the availability of free glucose in rumen which, in turn, promotes the growth of most bacteria, leading to greater production of volatile fatty acids like acetate, propionate and butyrate. Furthermore, Zhang et al.^33^ reported that acetate and propionate levels were affected in cows with high-yield compared to low-yield milk production. Furthermore, Shabat et al.^16^ reported that acetate and propionate concentrations were significantly higher in efficient animals compared to inefficient individuals. Ellis et al.^34^ and van Gastelen et al.^35^ positively associated acetate with phenotypic and environmental characteristics, such as methane emission (CH_4_). In this context, our study also indicated that these metabolites are of paramount importance for fecal Nelore steer metabolomics.

Recent studies have demonstrated a direct relationship between Nelore cattle rumen and fecal microbiomes^20,36^. In addition, the association between bacterial and fungal rumen, small intestine, cecum and feces microbiota and the feed efficiency phenotypes has also been reported for this breed^37^. Just as the fecal microbiome was studied and compared to the ruminal microbiome, the integration of metabolome data can also contribute to animal production. Interestingly, the representation of rumen metabolites in feces may assist and simplify future studies, in order to facilitate non-invasive field data collection. In other words, the fecal metabolome partially reflects the metabolic processes that occur in rumen and allows researchers to obtain information and, potentially, a phenotype description based on less invasive sampling procedures.

Another interesting observation arising from this study is the high glutamate concentrations in ruminal fluid and feces. Glutamate biosynthesis occurs from alpha-ketoglutarate, which may potentially use branched-chain amino acids, such as valine, leucine and isoleucine^38^. Thus, in the present study, glutamate synthesis in rumen may have contributed to the decreased availability of certain amino acids.

Furthermore, we observed that valine, pyruvate, glucose, lactate and ethanol concentrations were increased in the fecal samples. In rumen, the valine biogenesis uses pyruvate and isobutyrate as the main substrates^39^. Indeed, pyruvate is the product of glucose degradation by the glycolytic pathway^40,41^. In addition, pyruvate can be transformed into lactate by lactate dehydrogenase or converted into acetyl coenzyme-A and formate by pyruvate formate-lyase^42^, and, finally, acetyl-CoA can be reduced to ethanol. High concentrations of ethanol in the gastrointestinal tract have been reported as leading to significant consequences for the host^43^, although the ethanol concentrations observed herein were lower than reported as harmful to ruminants.

Succinate can be produced by many microorganisms in rumen, being this metabolite rapidly utilized by *Succiniclasticum* or metabolized by propionate-producing bacteria, which prevents its accumulation^44,45^. Accordingly, Xue et al.^41^ reported that decreased succinate concentrations can be explained by increased *Succiniclasticum*. Thus, in the present study increased succinate concentrations in feces may be due to the high production and reduced use of this compound in the rumen.

Xanthine, hypoxanthine, ornithine and uracil are intermediate degradation products of nucleic acids by rumen bacteria^46^. These compounds are rapidly degraded in rumen^47^, which may explain the lower concentrations of these metabolites in the feces. Alanine is also a product of the death of both gram-negative and gram-positive bacteria^48^, although the concentration of this compound was not significantly different between the assessed bio-samples.

The apolar metabolite results demonstrated a diversity of fatty acids in ruminal fluid and feces due to microorganism activity. Unsaturated fatty acids can have toxic effects for ruminal bacteria and decrease fiber digestibility^49,50^. Thus, despite the fact that fatty acids are not used as an energy source by microorganisms, a portion of the ruminal microbiota exhibits mechanisms to hydrolyze and biohydrogenate dietary lipids^51,52^. According to Saleem et al.^12^ and Bryszak et al.^53^, the dietary fatty acid composition also influences the biohydrogenation pathways performed by microbial population in the rumen. Therefore, the fatty acid flow leaving the rumen is higher than the dietary fatty acid intake^54^, and this process plays a vital role in energy production and storage, while also influencing lipid concentrations of final products, such as milk or meat^55^.

Stearic (C18:0) and palmitic acid (C16:0) were the most abundant apolar metabolites in the bio-samples assessed in the present study, and were reported as present at high concentrations in ruminal fluid by Saleem et al.^12^, Szczechowiak et al.^56^ and Bryszak et al.^53^. Palmitic acid is the end product of the biohydrogenation of palmitoleic acid (C16:1) although C16:0 also can be metabolized by rumen microbes, and thereby converted to other fatty acids^57^. Similarly, stearic acid is produced from the biohydrogenation of 18-carbon unsaturated fatty acids, like C18:2 and C18:3^5,58^, which are the main polyunsaturated fatty acids in ruminant diets, possibly supporting the results reported herein. In addition, stearic acid represents the majority of intestinal fatty acids in the rumen when an efficient biohydrogenation process takes place.

Our results also indicated that fatty acids concentrations in feces were higher than in the rumen. Individual fatty acids digestibility and absorption are not related to their intake, so it is possible that the amount of fatty acids reaching the duodenum may affect fatty acid solubility and incorporation into micelles, resulting in decreased digestibility and absorption. This may also be due to the microbial synthesis of fatty acids in the large intestine, which are probably not absorbed^54^.

The importance of fatty acids in the rumen and feces is positively related to feed efficiency phenotypes, as reported by Artegoitia et al.^6^, where a higher concentration of pentadecanoic acid (C15:0) was reported in steers with lower average daily gain. The reduction of methane emissions has also been associated to lauric (C12:0), myristic (C14:0) and linoleic (C18:2n6) acids in the review performed by Toprak et al.^59^. Furthermore, rumen fluid lipids have been almost exclusively applied to dairy cattle, with little research conducted on beef cattle. For this reason, easier and more practical strategies in obtaining the apolar metabolite information in feces can aid in understanding rumen lipid metabolism and improve animal production, as the fatty acids fecal content of Nelore exhibits a strong potential to reflect rumen digestion conditions.

The correlation network analysis was applied to compare the ruminal and fecal metabolic profiles shows that ruminal fluid exhibited correlations with 43 fecal metabolites, while feces exhibited correlations with 60 ruminal metabolites. These findings suggest that correlations are mainly centered in the uracil metabolite from ruminal fluid and feces samples from Nelore steers. Uracil is a nucleic acid base found in the RNA and in bacterial degradation products in the rumen. Increases in the concentrations of this metabolite in rumen have been observed when feeding bovines high-grain diets^11,33,43,46^. Increases have also been reported in the rumen of the high-yield dairy cows^32^ and in low residual feed-intake steers^60^. Uracil may, therefore, be an indicator for genetic microbial or plant material turnover in the rumen^60^, also increasing the synthesis and availability of crude microbial protein^30,61,62^. Consequently, this metabolite seems to have a significant impact on the biodiverse ruminal ecosystem, directly reflecting the metabolic feces profile.

One of the most interesting observations from this study is that the different metabolites detected in ruminal fluid and feces were specifically correlated to the same metabolic pathways as verified by the pathway impact analysis. In summary, we have characterized the ruminal and fecal metabolome biodiversity of Nelore for the first time. This study offers a new comprehensive insight into the biochemical mechanisms of ruminal fluid and its association with fecal samples, and also demonstrates that most metabolites are common to both environments. In general, these data emphasize the importance of gaining a better understanding of the biochemical functions of the bovine gastrointestinal tract. In addition, although our findings have shown a close relationship between the metabolic ruminal profile and feces in the Nelore breed. Additional studies that promote alterations in rumen are required to verify whether these modifications are also reflected in the feces and provide insights into animal production strategies.

## Material and methods

### Production of experimental animals and sample collection

All experimental procedures were conducted in accordance with animal welfare and humane slaughter guidelines and were approved by the EMBRAPA Livestock Science Ethics Committee on Animal Experimentation, São Carlos, São Paulo (Protocol No. 09/2016).

A population of 26 contemporaneous uncastrated Nelore steers (*Bos indicus*) was placed in an experimental feedlot. Animals averaging 329.5 ± 34.2 kg of initial body weight and aged 20–21 months old were allocated to two collective pens containing 13 animals/pen for 105 d, of which the first 15 were exclusively for animal adaptation, followed by the growth and finishing stages. The animals received a diet consisting of corn silage (72.8%), soybean meal (3.06%), corn grains (21.4%), protected fat (1.19%), urea (0.59%) and Confinatto N235 Agroceres Multimix® (0.91%) twice a day.

After the finishing phase, fecal samples of the rectal ampulla were collected from each animal, kept on ice for approximately 2 h and stored at -80 °C for the metabolomic assays. The animals were then sent for slaughter at a final weight of approximately 477.3 ± 41.5 kg at 23–24 months of age, in accordance to the Humane Slaughter of Cattle guidelines. During the slaughter, the ruminal fluid of each animal was also collected, immediately immersed in liquid nitrogen and then stored at − 80 °C for the metabolomic assays.

### Untargeted metabolomics analysis

#### Sample preparation of polar metabolites

Rumen fluid samples of the 26 animals were thawed on ice for approximately 2 h and centrifuged at 13,000 × g (10 min at 4 °C). The supernatants were collected and re-centrifuged for particulate material sedimentation. Next, 400 µL of the centrifuged ruminal liquid was solubilized in 200 µL of deuterium oxide phosphate buffer (0.10 M, pD = 7.4) containing 0.050% w/w of sodium 3-trimethylsilyl-2,2,3,3-d4-propionate (TMSP-d4, SigmaAldrich) and 0.02% m/v of sodium azide. Finally, 600 µL were transferred to a 5 mm NMR tube.

Fecal samples (~ 300 mg) were extracted directly using 900 µL of deuterium oxide phosphate buffer (0.10 M, pD = 7.4) containing 0.050% w/w of sodium 3-trimethylsilyl-2,2,3,3-d4-propionate (TMSP-d4, SigmaAldrich). Samples were vortexed for 60 s and centrifuged at 13,000 × *g* (10 min at 4 °C). The supernatant was then collected in a new microtube, re-diluted with deuterium oxide (3:1 v/v) and 600 µL were transferred to a 5 mm NMR tube. Two quality control (QC) samples constituted by a pool of aliquots of the metabolic extracts of rumen liquid and feces representing each condition.

### ^1^H NMR spectrum acquisition

All ^1^H NMR spectra for the rumen and fecal samples were acquired at 298 K on a 14 T Bruker Avance III spectrometer (Bruker BioSpin, Rheinstetten, Germany) equipped with a 5 mm PABBO probe head with gradients, automated tuning and matching accessory (ATMATM), BCU-I for temperature regulation and a Sample-Xpress sample changer. The ^1^H spectra were acquired using 1D NOESY-presaturation pulse sequence (Bruker 1D noesygppr1d), 64 K data points, with a spectral width of 20.0276 ppm, an acquisition time of 2.726 s, a recycle delay of 4 s, relaxation delay of 4 s, dummy scans of 4, an accumulation of 256 transients, and a mixing time of 0.005 s. FIDs were multiplied by a 0.3 Hz exponential multiplication function prior to Fourier transformation. Phase and baseline correction were applied, the TMSP-d4 signal was calibrated at δ 0.00 ppm, and the integrals of the spectral signal areas were determined using the Topspin Version 3.6 software for NMR analysis (Bruker Inc., Karlsruhe, Germany). The 2D NMR experiments as ^1^H–^1^H *J*‐resolved acquisition parameters were as follows: number of data points, 8192 for F2 and 128 for F1; spectral width, 6944.4 Hz for F2 and 6944.4 Hz for F1; relaxation delay, 2 s; number of scans, 64; number of dummy scans, 16; acquisition time, 0.589 s for F2 and 0.009 s for F1. The ^1^H–^13^C heteronuclear single quantum coherence (HSQC) acquisition parameters were as follows: number of data points, 2048 for F2 and 256 for F1; spectral width, 7812.5 Hz for F2 and 24,900.8 Hz for F1; relaxation delay, 1.5 s; number of scans, 64; number of dummy scans, 32; acquisition time, 0.131 s for F2 and 0.005 s for F1.

### Polar metabolite identification and quantification

The resulting ^1^H NMR spectra were processed and analyzed using the Chenomx NMR Suite Professional software package version 6.0 (Chenomx Inc., Edmonton, AB, Canada). The identified metabolites were also defined based on public databases, e.g., the Bovine Metabolome Database (BMDB, http://www.cowmetdb.ca), Human Metabolome Database (HMDB, http://www.hmdb.ca) and the Biological Magnetic Resonance Data Bank (BMRB, www.bmrb.wisc.edu). For quantification, individual metabolite peaks were integrated and quantified relative to the “Electronic REference To access in vivo Concentrations 2” (ERETIC2) signal experiment, performed using a 2 mM Sucrose standard, while the fixed receiver gain (RG) was used for the samples.

### Sample preparation of apolar metabolites

The total fat content of ruminal fluid and feces were determined according to Folch method^63^ with few modifications. In brief, 500 µL of ruminal fluid or 500 mg of feces were used for lipid extraction with chloroform and methanol (2:1 v/v). After the extraction procedure, the samples were centrifuged at 13,000 × *g*, organic phase was collected, the solvent was evaporated under a gentle nitrogen flow and the total weight of lipids was estimated gravimetrically. Extracted lipids were transmethylated to fatty acid methyl ester (FAME) using boron trifluoride (BF_3_) in methanol according to Joseph and Ackman^64^.

### Apolar metabolites detection by Gas chromatography flame ionization (GC-FID)

FAME were analyzed using an GC-2014 (Shimadzu, Kyoto, Japan) gas chromatograph equipped with a flame ionization detector (FID), AOC-20 Auto Injector (Shimadzu, Kyoto, Japan) and a capillary column SUPELCOWAX® 10 (I.D. 30 m × 0.25 mm, df 0.25 μm, Sigma-Aldrich, St. Louis, MO). The chromatographic conditions were as follows: injector at 250 °C, injected volume 1 µL with split ratio set to 1:20, and the carrier gas was helium at 1.0 mL min^−1^. The column oven was programmed as follows: 150 °C for 5 min, followed by an increment of 10 °C min^−1^ to 250 °C hold for 10 min. FID detector operated at 260 °C. The quantification (mg g^−1^ of total lipids) was performed using the internal standard method (C23:0) as described by Visentainer^65^. Biological samples were analyzed in technical triplicate.

### Statistical analyses

The polar and apolar metabolites were analyzed by MIXED procedure available in the SAS statistical program (SAS Institute, Cary, NC, USA, 2011). The statistical model included pen as fixed effect and initial and final weight of feedlot as covariates. The metabolites results were deemed significant when *P* < 0.05. The obtained ^1^H NMR data had a binning of 0.04 ppm applied and were transformed into a data matrix using the MNova software. Next, data were analyzed in the MetaboAnalyst 4.0 platform (http://www.metaboanalyst.ca), using a Principal Component Analysis (PCA) and Partial Least Squares Discriminant Analysis (PLS-DA). Data preprocessing enrolled data no sample normalization, and the Pareto scaling was used (mean-centered and divided by the square root of standard deviation of each variable). Principal component was used for the discrimination of the analyzed ruminal fluid and feces samples. Specifically, for the PLS-DA, LOOCV was applied as the cross-validation method (Supplementary Fig. 1). The accuracy and variable importance in projection (VIP) were also assessed to determine the performance and importance features of the analysis, respectively. Spearman’s correlation analysis of ruminal and fecal metabolites was performed using the Cytoscape software (http://www.cytoscape.org). The correlation results were plotted when *P* < 0.05. Based on the Kyoto Encyclopedia of Genes and Genomes (KEGG, http://www.kegg.jp) network diagrams were construct between metabolites and the impact factor of the topology analyses of metabolic pathways was graphically presented using MetaboAnalyst 4.0^66^.

## Supplementary Information

Supplementary Information 1.

Supplementary Information 2.
