# Supplementary material for: Comparative untargeted metabolome analysis of ruminal fluid and feces of Nelore steers (Bos indicus)
Source: Sci Rep. 2021 Jun 17;11:12752. doi: 10.1038/s41598-021-92179-y (PMC8211696; doi:10.1038/s41598-021-92179-y)
Supplement: Supplementary file 1 — Supplementary Information 1. [file 41598_2021_92179_MOESM1_ESM.docx]

**
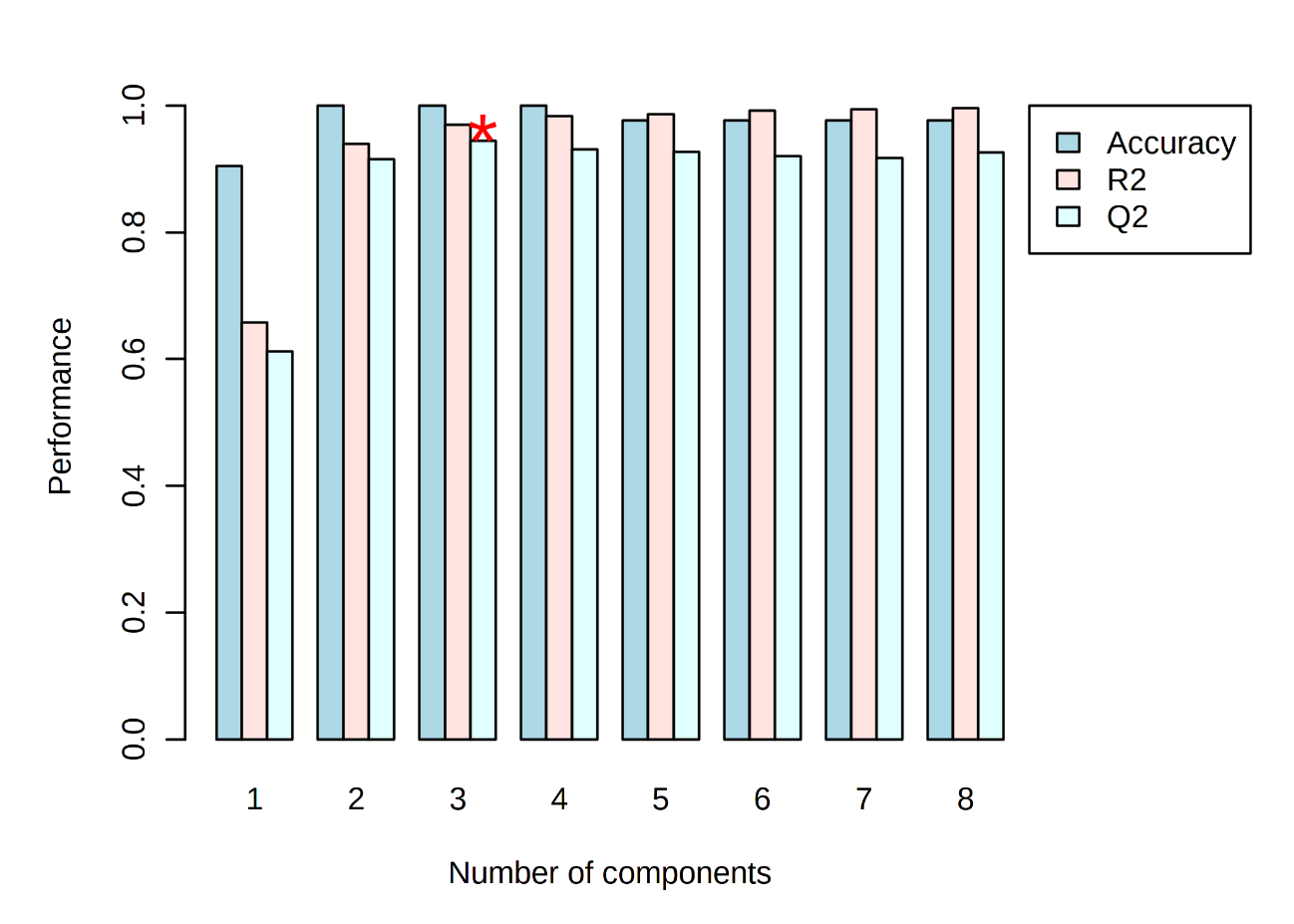
Supplementary Figure 1.** Cross validation of the PLS-DA models. Bar plots showing the three performance measures (prediction accuracy, R^2^ and Q^2^) using different number of components. The red * indicates the best values of the currently selected measures (Q^2^).
